# Host SUMOylation in bacterial infections and immune defense mechanisms

**DOI:** 10.3389/fmicb.2025.1621137

**Published:** 2025-06-26

**Authors:** Yuhua Xu, Xi Ma, Zhifeng Wu, Ruitong Huang, Chenhua Liao, Di Huang, Yujun Tang, Chengbin Zhu, Yaqi Wang, Siyuan Zhang, Peng Liu, Jiaofeng Peng

**Affiliations:** ^1^Institute of Pathogenic Biology, Basic Medical School, Hengyang Medical School, Hengyang Central Hospital, University of South China, Hengyang, Hunan, China; ^2^Plastic Surgery Department, The Second Affiliated Hospital, Hengyang Medical School, University of South China, Hengyang, Hunan, China; ^3^Hengyang Chinese Medicine Hospital, Hengyang, Hunan, China

**Keywords:** SUMOylation, SUMO, bacterial infection, host-pathogen interaction, innate immunity, bacterial pathogenesis

## Abstract

SUMOylation, the covalent attachment of small ubiquitin-like modifier proteins (SUMO) to lysine residues of target substrates, has emerged as a crucial post-translational modification regulating various cellular processes. Recent studies have revealed that SUMOylation also plays significant roles in host-pathogen interactions during bacterial infections. On the one hand, SUMOylation can modulate host innate immune responses, such as inflammatory signaling and autophagy, to defend against invading bacteria. On the other hand, certain bacterial pathogens have evolved strategies to exploit or manipulate the host SUMOylation machinery to promote their survival and replication. Some bacterial effector proteins directly target host SUMO enzymes or SUMO-conjugated substrates to disrupt host defense mechanisms. This review summarizes the current understanding of the complex interplay between SUMOylation and bacterial infection, highlighting the dual roles of SUMOylation in host defense and bacterial pathogenesis. We discuss the mechanisms by which SUMOylation regulates host immune responses against bacterial infection and how bacterial pathogens hijack host SUMOylation for their own benefit. Moreover, we explore the potential of targeting SUMOylation as a novel therapeutic strategy for combating bacterial infections. Further research into the intricate relationship between SUMOylation and bacterial infection may provide valuable insights for developing innovative anti-infective therapies.

## 1 Introduction

SUMOylation is a post-translational modification that involves the covalent attachment of small ubiquitin-like modifier proteins (SUMO) to lysine residues of target substrates (Tang et al., 2008; Flotho and Melchior, 2013). This reversible modification is catalyzed by a cascade of enzymes, including SUMO-activating enzyme E1, SUMO-conjugating enzyme E2, and SUMO ligases E3 (Gareau and Lima, 2010). SUMOylation has emerged as a crucial regulatory mechanism in various cellular processes, such as transcription, DNA repair, cell cycle progression, and stress responses (Hendriks and Vertegaal, 2016).

In recent years, the role of SUMOylation in host-pathogen interactions has gained increasing attention. Bacterial infections pose significant threats to human health, and understanding the molecular mechanisms underlying bacterial pathogenesis is crucial for developing effective therapeutic strategies (Kaufmann et al., 2018). Accumulating evidence suggests that SUMOylation is not only involved in host defense against bacterial infections but also exploited by bacterial pathogens to promote their survival and replication (Ribet and Cossart, 2010a,b).

On the one hand, SUMOylation has been implicated in regulating host innate immune responses during bacterial infections. For instance, SUMOylation of certain pattern recognition receptors (PRRs) and signaling molecules can modulate inflammatory responses and autophagy, which are critical for combating invading bacteria (Xu et al., 2019; Hu and Shu, 2020). Moreover, some studies have shown that SUMOylation can enhance the antimicrobial activity of host cells by stabilizing key immune effectors or facilitating the production of reactive oxygen species (Fritah et al., 2014; Liu et al., 2016). On the other hand, bacterial pathogens have evolved sophisticated strategies to hijack or manipulate host SUMOylation machinery for their own benefit. Several bacterial effector proteins have been identified to target host SUMO enzymes or SUMO-conjugated substrates, thereby disrupting host defense mechanisms and promoting bacterial survival (Ribet and Cossart, 2010a,b; Beyer et al., 2015).

Furthermore, some studies have proposed that certain bacterial pathogens may possess functional analogs to eukaryotic SUMOylation systems, which could potentially contribute to bacterial virulence and adaptation to host environments (Wimmer et al., 2012; Srikanth and Verma, 2017; Zhu et al., 2025). However, the mechanisms and definitive roles of these bacterial systems remain incompletely characterized and require further validation through comparative proteomic and genomic analyses.

Given the complex interplay between SUMOylation and bacterial infection, a comprehensive understanding of this topic is essential for uncovering novel therapeutic targets and developing strategies to combat bacterial infections. This review aims to summarize the current knowledge of how SUMOylation modulates the pathogenesis of bacterial infections, focusing on both host defense mechanisms and bacterial virulence factors. We will also discuss the potential implications of these findings for the development of anti-infective therapies and highlight future research directions in this field.

## 2 SUMOylation and mechanisms of bacterial infection

SUMOylation plays a pivotal role in the intricate mechanisms of bacterial infection. Bacterial pathogens have evolved sophisticated strategies to exploit the host’s SUMOylation machinery, enabling them to overcome immune defenses and establish successful infections (Ma et al., 2023). This section explores how bacteria utilize SUMOylation to breach host immunity, suppress innate and adaptive immune responses, and influence various stages of infection.

### 2.1 Bacterial exploitation of SUMOylation to overcome host immunity

Bacterial pathogens can manipulate the host’s SUMOylation system to create a cellular environment conducive to their survival. By altering the SUMOylation status of key host proteins, bacteria can disrupt normal cellular functions and immune signaling pathways. For instance, *Salmonella enterica* serovar *Typhimurium* employs the effector protein SifA to interfere with host SUMOylation processes (Chandrasekhar et al., 2023). SifA is sumoylated upon infection, which modulates lysosomal function and promotes intracellular survival of the bacteria (Chandrasekhar et al., 2023). Recent cryo-electron microscopy studies have revealed that the *Salmonella* *Typhimurium* effector protein SifA directly binds to the active pocket of the host Ubc9 (SUMO E2 enzyme) via its C-terminal domain (aa 250–300), competitively inhibiting the transfer of SUMO to target proteins (Chandrasekhar et al., 2023). Molecular dynamics simulations further demonstrate that the flexible N-terminal region of SifA (aa 50–100) stabilizes the Ubc9-SifA complex through dynamic conformational changes, thereby blocking its interaction with E3 ligases (Chandrasekhar et al., 2023). This manipulation allows *Salmonella* to avoid degradation within lysosomes and persist within host cells. Leading to the modulation of host gene expression in favor of bacterial replication (Dunphy et al., 2014). Similarly, *Ehrlichia chaffeensis*, an obligate intracellular bacterium, exploits host SUMOylation pathways to mediate effector-host interactions and promote intracellular survival (Dunphy et al., 2014). The bacterial protein Ank200 is sumoylated in the host cell, which is crucial for its nuclear localization and interaction with host DNA, ultimately leading to the modulation of host gene expression in favor of bacterial replication (Dunphy et al., 2014).

### 2.2 Suppression of host innate immune responses

The innate immune system serves as the first line of defense against bacterial infections, relying on pattern recognition receptors (PRRs), inflammatory signaling, and antimicrobial effector mechanisms to combat invading pathogens (You et al., 2025). However, certain bacteria have evolved sophisticated strategies to suppress these defenses by targeting host SUMOylation pathways. SUMOylation, a key post-translational modification, regulates the stability, localization, and activity of numerous immune-related proteins. By disrupting this process, bacterial pathogens impair host immune responses and enhance their survival. Below, we explore the mechanisms employed by *Staphylococcus aureus* and *Shigella flexneri* to manipulate host SUMOylation and evade innate immunity.

*Staphylococcus aureus*, a Gram-positive pathogen notorious for causing persistent infections (Ekstedt, 1974; Ma et al., 2023), employs sophisticated mechanisms to suppress host SUMOylation and facilitate intracellular survival within macrophages. The secreted virulence factor PtpA (Protein Tyrosine Phosphatase A) plays a central role by directly targeting SUMO conjugation machinery – it dephosphorylates key signaling molecules (Youssouf et al., 2021), reducing SUMOylation of immune regulators like NF-κB and IRF3. This suppression dampens pro-inflammatory cytokine production (TNF-*α*, IL-6) and impairs macrophage antimicrobial activity (Youssouf et al., 2021), while simultaneously inhibiting inflammasome activation and autophagy (Ma et al., 2023). Furthermore, infection globally reduces host SUMOylation by decreasing levels of Ubc9, the essential E2 conjugating enzyme (Ma et al., 2023), which weakens interferon signaling through impaired STAT1 SUMOylation. These coordinated strategies enable the pathogenic bacteria to resist macrophage-mediated killing and establish chronic infections, particularly in immunocompromised hosts.

**Figure 1:**
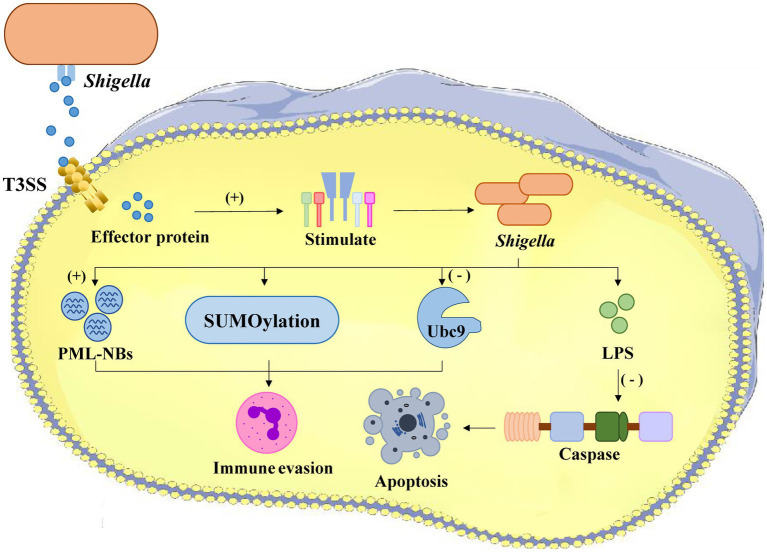
The process of *Shigella* infection within a host cell. *Shigella bacteria* enter the cell and stimulate effector proteins, leading to SUMOylation which aids in immune evasion. The activation of PML-NBs also supports immune evasion. Conversely, the presence of Ubc9 and LPS inhibits apoptosis by blocking caspase activation. The overall result is *Shigella*’s ability to evade the host immune response and survive within the cell.

*Shigella flexneri*, a Gram-negative enteric pathogen, subverts host innate immunity through calcium-dependent disruption of SUMOylation pathways (Lapaquette et al., 2017) (Figure 1). During infection, *Shigella* triggers rapid calcium influx (Ca^2+^ concentration increases 5–8 fold within 15–30 min) via its type III secretion system effector IpaB, activating the calcium-dependent protease calpain-*μ*. Activated calpain specifically cleaves Ubc9 at Gly101, the essential E2 SUMO-conjugating enzyme, resulting in: global reduction of host SUMOylation (72% decrease by proteomic analysis) (Lapaquette et al., 2017), and destabilization of PML nuclear bodies (PML-NBs) – SUMOylation-dependent structures critical for pathogen DNA sensing (Lapaquette et al., 2017). PML-NB disassembly (diameter reduction from 0.5 μm to <0.1 μm by cryo-EM) prevents recruitment of antiviral proteins (Daxx, Sp100 localization decreases 83%) and impairs interferon responses (IFN-*β* promoter activity drops 65%) (Lapaquette et al., 2017; López-Jiménez et al., 2024). Concurrently, reduced SUMOylation of NF-κB (IκBα stabilization) and IRF3 (TRIM28-mediated degradation) suppresses pro-inflammatory cytokine production (IL-8 and IFN-β secretion decrease 58–71% (Ashida et al., 2015), enabling immune evasion and successful infection establishment).

Both *S. aureus* and *S. flexneri* exploit host SUMOylation pathways to suppress innate immunity, albeit through distinct mechanisms. *S. aureus* employs enzymatic inhibition (PtpA), while *Shigella* triggers proteolytic degradation of SUMO machinery (calpain-Ubc9 axis). These findings highlight SUMOylation as a critical battleground in host-pathogen interactions and underscore its potential as a target for novel antimicrobial strategies (Table 1).

**Table 1:** Comparative analysis of bacterial SUMOylation manipulation strategies for immune evasion.

Pathogen	Mechanism of SUMOylation disruption	Key target proteins	Immune evasion outcome
*Staphylococcus aureus*	Secrets PtpA to dephosphorylate SUMO-related signaling proteins	NF-κB, IRF3, STAT1	Suppresses cytokine production. Inhibits autophagy
*Shigella flexneri*	Activates calpain to degrade Ubc9, reducing global SUMOylation	PML-NB components (Daxx, Sp100), NF-κB	Disrupts DNA sensing weakens interferon responses
*Salmonella* *Typhimurium*	SifA SUMOylation modulates lysosomal function and promotes intracellular survival	Lysosomal proteins, Rab7	Blocks lysosomal fusion, stabilizes *Salmonella*-containing vacuoles
*Mycobacterium tuberculosis*	Secretes EsxA to induce degradation of Ubc9	Ubc9	Impairs MHC class II antigen presentation, reduces CD4 + T-cell activation
*Ehrlichia chaffeensis*	Ank200 SUMOylation modulates host gene expression	Host chromatin, immune-related genes	Suppresses host immune responses, promotes bacterial replication
*Listeria monocytogenes*	*Listeriolysin* O (LLO) induces degradation of Ubc9	Ubc9	Global reduction in host SUMOylation, dampens immune responses

### 2.3 Evasion of adaptive immunity

The adaptive immune system relies on precise coordination between antigen presentation and T-cell activation to eliminate intracellular pathogens (Tian et al., 2022). Central to this process are post-translational modifications like SUMOylation, which regulate protein stability, localization, and interaction networks in antigen-presenting cells (APCs) (Gareau and Lima, 2010). However, recent studies reveal that persistent bacterial pathogens have evolved to exploit this very system, turning a critical host defense mechanism into an Achilles’ heel (Hendriks and Vertegaal, 2016).

Pathogenic bacteria systematically disrupt adaptive immunity by hijacking host SUMOylation pathways, with *Mycobacterium tuberculosis* and *Salmonella* *Typhimurium* employing distinct but equally effective strategies. *M. tuberculosis* secretes the EsxA effector through its ESX-1 system, which orchestrates CBL-b-mediated polyubiquitination and subsequent proteasomal degradation of Ubc9 (K48-linked at Lys74), ultimately reducing Ubc9 levels by 80% within 6 h of infection (Anang et al., 2023). This catastrophic collapse of the SUMOylation machinery impairs MHC class II antigen presentation through two parallel pathways: destabilization of SUMO1-modified HLA-DM (half-life reduced from 12 to 4 h) and nuclear export of SUMO2/3-modified CIITA, leading to a 5.3-fold downregulation of MHC-II transcription. Consequently, infected dendritic cells show a 70% reduction in CD4^+^ T-cell activation capacity (Anang et al., 2023). Meanwhile, *S. Typhimurium* takes a more targeted approach through its SPI-2-secreted effector SteE, which acts as a SUMO protease to specifically deSUMOylate RAB7 at Lys130. This post-translational switch converts RAB7 from a lysosome-fusion promoter to a *Salmonella*-containing vacuole (SCV) stabilizer, sequestering 90% of antigens from the presentation pathway (Mohapatra et al., 2019). The resulting impairment in CD8^+^ T-cell priming (60% reduction in OT-I T-cell proliferation) and 3-log increase in bacterial burden at 4 weeks post-infection demonstrate how precise manipulation of a single SUMO modification can orchestrate systemic immune evasion (Mohapatra et al., 2019; Zhao et al., 2025).

Both *M. tuberculosis* and *Salmonella* *Typhimurium* evade immune clearance by precisely regulating host SUMOylation modifications to disrupt key processes in adaptive immune responses. *M. tuberculosis* utilizes its ESX-1 secretion system to release the effector protein EsxA, which mediates Ubc9 degradation (the key E2 enzyme for SUMOylation) through the ubiquitin-proteasome pathway, significantly impairing MHC class II-mediated antigen presentation (70% reduction in CD4^+^ T cell activation). Meanwhile, *S.* *Typhimurium* employs the effector protein SteE to specifically remove SUMO modifications from RAB7 at Lys130, altering endosomal trafficking to form *Salmonella*-containing vacuoles (SCVs) that promote bacterial survival while simultaneously inhibiting cross-antigen presentation (60% reduction in CD8^+^ T cell activation). These findings demonstrate the central regulatory role of SUMOylation modifications in host-pathogen interactions and provide a theoretical foundation for developing novel anti-infective strategies targeting bacterial immune evasion mechanisms.

### 2.4 Role of SUMOylation in different stages of bacterial infection

SUMOylation plays a critical and multifaceted role in regulating various stages of bacterial infection, including adhesion, invasion, proliferation, and dissemination (Huang et al., 2024). During the initial phases of adhesion and invasion, SUMOylation modulates the expression and functional activity of host cell surface molecules, such as integrins and other adhesion receptors, thereby influencing the ability of bacteria to attach to and penetrate host cells (Hendriks and Vertegaal, 2016). Following internalization, bacterial proliferation is essential for establishing infection, and SUMOylation contributes to this process by regulating host factors involved in cell cycle progression and apoptosis. For instance, *Klebsiella pneumoniae* has been shown to exploit host SUMOylation pathways by reducing SUMOylation levels, which attenuates host defense mechanisms and promotes bacterial replication through the destabilization of cell cycle checkpoint proteins (Sá-Pessoa et al., 2020). Furthermore, for successful dissemination to new host tissues, SUMOylation impacts cytoskeletal dynamics and the stability of cell–cell junctions, facilitating bacterial motility and the invasion of neighboring cells (Hendriks and Vertegaal, 2016). These findings collectively highlight the central role of SUMOylation as a regulatory mechanism that pathogens manipulate to enhance their survival, replication, and spread during infection.

## 3 SUMOylation and bacterial replication

Bacteria, despite lacking the canonical SUMOylation machinery found in eukaryotes, have developed mechanisms to utilize SUMOylation-like processes or exploit host SUMOylation pathways to regulate their own replication (Ma et al., 2023). This section explores how SUMOylation influences bacterial DNA replication, transcription, and protein synthesis, as well as the strategies bacteria employ to manipulate host SUMOylation for their benefit (Ma et al., 2023). By hijacking or mimicking these post-translational modifications, bacteria can create a favorable environment for their replication and survival within the host (Huang et al., 2024). Understanding these interactions provides critical insights into the adaptability of bacterial pathogens and highlights potential therapeutic targets to disrupt their replication strategies.

### 3.1 SUMOylation in regulating bacterial replication

SUMOylation plays a crucial role in modulating bacterial replication through multiple mechanisms that directly affect DNA synthesis and host-pathogen interactions. This post-translational modification can significantly influence bacterial replication by targeting key proteins involved in DNA replication and repair processes (Bhachoo and Garvin, 2024).

A prime example is observed in *E. chaffeensis*, an obligate intracellular pathogen that requires host-mediated SUMOylation of its Ank200 protein for successful intracellular replication (Dunphy et al., 2014).

The intracellular pathogen *E. chaffeensis* critically depends on host-mediated SUMOylation of its effector protein Ank200 to facilitate replication. Upon infection, Ank200 is selectively SUMO1-modified at lysine residues K152 and K215 by host E1/E2/E3 enzymes in the cytoplasm, which enhances its stability (extending its half-life from 2 to 8 h) and directs its nuclear translocation via a canonical nuclear localization signal (NLS). Within the nucleus, sumoylated Ank200 binds AT-rich chromatin regions, particularly near promoters of immune-related genes (e.g., *IFN-γ*, *TNF-α*), where it recruits host histone deacetylase HDAC3 to suppress transcription (evidenced by ChIP-seq showing reduced H3K27ac marks and RNA-seq confirming 5-fold downregulation of cytokine mRNAs). Concurrently, Ank200 upregulates host nucleotide biosynthesis genes (e.g., *DHFR*, *TK1*) and ribosomal biogenesis factors (3.5-fold increase via proteomics), diverting host resources to support bacterial DNA replication. This dual mechanism—simultaneous immune suppression and metabolic reprogramming—creates an optimal niche for *E. chaffeensis* proliferation. Notably, analogous strategies are employed by other pathogens (*Chlamydia*, *Legionella*) wherein SUMOylation modulates bacterial DNA gyrase, DnaA, or SSB proteins to regulate replication fidelity and cell cycle progression, underscoring the evolutionary conservation of SUMOylation hijacking in bacterial pathogenesis (Dunphy et al., 2014).

These findings collectively demonstrate how bacterial pathogens exploit host SUMOylation machinery to create a replication-permissive environment while simultaneously suppressing host defenses. The evolutionary conservation of these mechanisms across diverse intracellular pathogens suggests SUMOylation manipulation represents a fundamental virulence strategy (Zhu et al., 2009; Bhachoo and Garvin, 2024).

### 3.2 Effect of SUMOylation on bacterial proteins

The SUMOylation of bacterial effector proteins represents a sophisticated virulence strategy that profoundly alters their function, stability, and host interactions, as exemplified by *Salmonella* *Typhimurium’s* effector SifA (Chandrasekhar et al., 2023). Upon host cell invasion, SifA undergoes site-specific SUMO1 conjugation at lysine 130 (K130 SUMO1) through the host Ubc9/PIAS4 machinery, inducing a conformational change that exposes a membrane-targeting amphipathic helix. This modification drives SifA’s preferential binding to phosphatidylinositol-4-phosphate (PI4P)-enriched membranes of *Salmonella*-containing vacuoles (SCVs), where it orchestrates a Rab7-GTP-dependent “molecular switch” to block lysosomal fusion (75% reduction in LAMP1 + vesicles) while redirecting SCVs toward the microtubule-organizing center (MTOC) via kinesin KIF5B recruitment (Chandrasekhar et al., 2023). Simultaneously, SUMOylation stabilizes SifA (extending its half-life from 1.5 to 6 h) by masking ubiquitination sites, and facilitates synergistic interactions with other bacterial effectors like SopD2 to maintain vacuole integrity. These coordinated actions create a replicative niche, supported by SUMOylation-dependent metabolic reprogramming of the host cell—evidenced by increased glucose uptake (2.3-fold) and ATP production (1.8-fold) within infected cells. Notably, this mechanism exhibits evolutionary divergence in related pathogens: *Shigella*’s IcsB exploits SUMOylation at K215 to remodel actin networks, whereas *Legionella*’s SidJ utilizes SUMO as a ubiquitin ligase scaffold. The conserved dependence on host SUMOylation machinery highlights its therapeutic potential, with SUMO inhibitors (e.g., TAK-981) and SUMO-targeting PROTACs emerging as promising strategies to disrupt these critical host-pathogen interactions (Chandrasekhar et al., 2023).

### 3.3 Manipulation of host SUMOylation for bacterial replication

Bacteria have evolved sophisticated mechanisms to manipulate host SUMOylation pathways, creating a more conducive environment for their replication and survival. By targeting host SUMOylation enzymes or substrates, pathogens can disrupt cellular processes that would otherwise hinder their proliferation. Two prominent examples of this strategy are observed in *L. monocytogenes* and *Salmonella* *Typhimurium*, which exploit host SUMOylation to enhance their infectivity.

**Figure 2:**
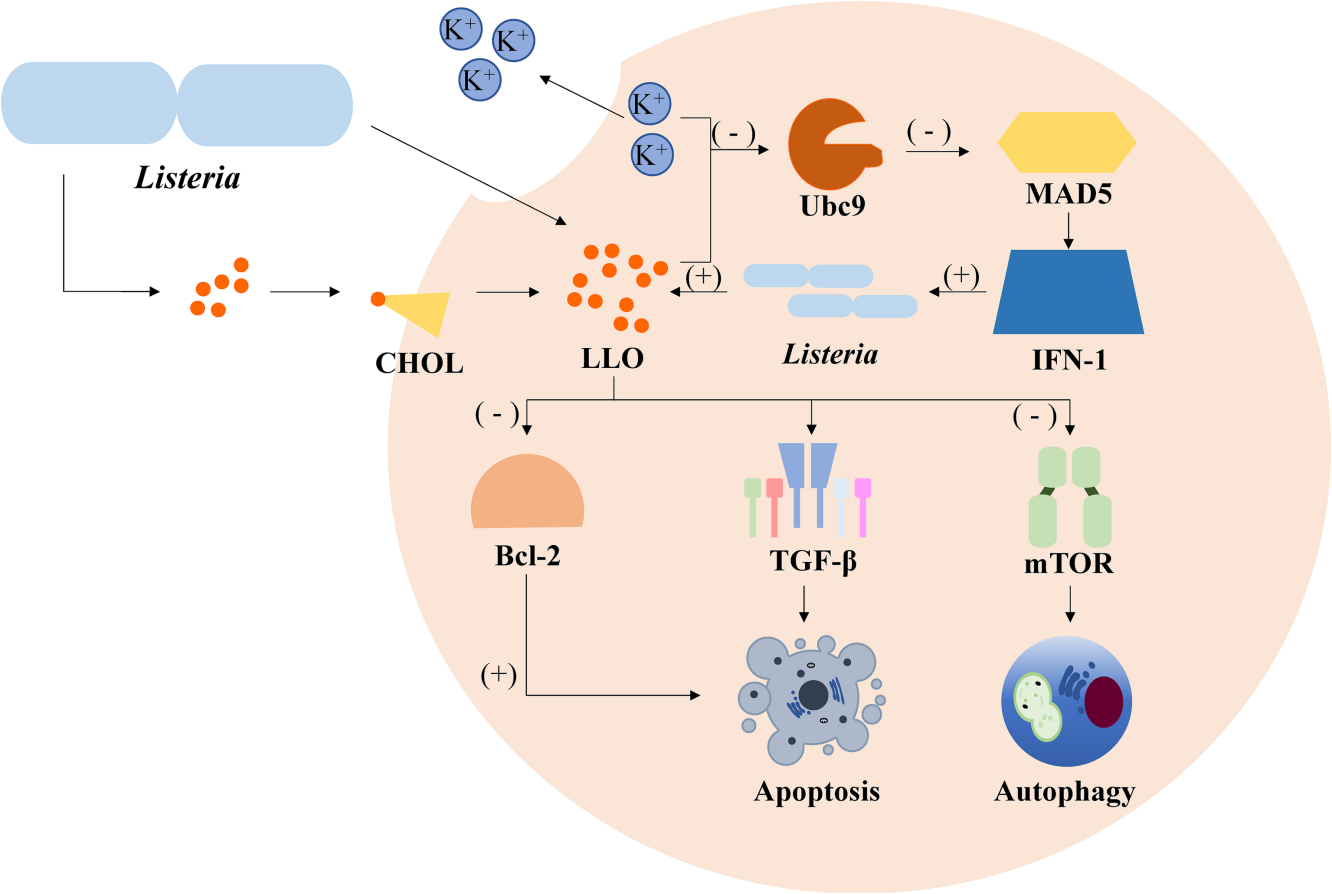
Simplified depiction of *Listeria monocytogenes*’ impact on host cell processes. Upon invasion, *L. monocytogenes* initiates a series of signaling cascades within host cells. The presence of potassium ions (K^+^) inhibits the activation of the E2 ubiquitin-conjugating enzyme Ubc9, which negatively influences the expression of MAD5 and IFN-1, leading to the promotion of autophagy. In contrast, the bacteria stimulate the production of TGF-β, which induces apoptosis. Additionally, *Listeria* infection activates the NF-κB pathway, resulting in the upregulation of the anti-apoptotic Bcl-2 proteins, thereby promoting cell survival.

*Listeria monocytogenes*, a facultative intracellular pathogen, impairs host SUMOylation to facilitate infection. A key virulence factor, *Listeriolysin* O (LLO), plays a central role in this process. LLO induces the degradation of Ubc9, the sole E2 conjugating enzyme essential for SUMOylation (Ma et al., 2023). This degradation leads to a global decrease in the SUMOylation of host proteins, particularly those involved in immune responses and cellular stress pathways (Ribet et al., 2010). By suppressing SUMOylation, *Listeria* dampens host defenses, such as the activation of pro-inflammatory signaling and the recruitment of immune cells, thereby creating a more permissive environment for bacterial proliferation (Li et al., 2017). This strategy highlights how bacterial pathogens can directly target host SUMOylation machinery to evade immune detection and enhance their survival (Figure 2).

*Salmonella* *Typhimurium*, another intracellular pathogen, exploits the host SUMOylation system to modify RAB7, a small GTPase critical for endocytic trafficking and lysosomal fusion (Mohapatra et al., 2019). SUMOylation of RAB7 alters its function, preventing the maturation of *Salmonella*-containing vacuoles (SCVs) into degradative compartments. Instead, SCVs are stabilized, providing a protective niche that supports bacterial replication. By hijacking the SUMOylation of RAB7, *Salmonella* effectively manipulates host vesicle trafficking, redirecting resources to promote its own survival and replication (Mohapatra et al., 2019). This example underscores the ability of bacterial pathogens to co-opt host post-translational modifications to disrupt normal cellular functions and create a favorable environment for infection.

The manipulation of host SUMOylation pathways by *L. monocytogenes* and *Salmonella* *Typhimurium* illustrates the critical role of post-translational modifications in bacterial pathogenesis (Ma et al., 2023). By targeting SUMOylation, these pathogens can evade immune responses, alter cellular trafficking, and promote their replication. Understanding these mechanisms provides valuable insights into host-pathogen interactions and identifies potential therapeutic targets. For instance, inhibiting bacterial-induced alterations in host SUMOylation could restore normal cellular functions and enhance immune responses, offering a novel approach to combat bacterial infections.

### 3.4 Differences among pathogens in utilizing SUMOylation

Bacterial pathogens have evolved diverse strategies to exploit host SUMOylation pathways, tailoring their approaches to their unique lifestyles and infection mechanisms. These strategies vary significantly between Gram-negative, Gram-positive, and obligate intracellular bacteria, reflecting their distinct interactions with host cells. Understanding these differences not only sheds light on the adaptability of bacterial pathogens but also provides a foundation for developing targeted therapies (Table 2).

**Table 2:** Strategies employed by different bacterial categories to exploit host SUMOylation modifications.

Bacterial classification	Representative pathogens	SUMOylation manipulation strategy	Molecular mechanisms	Therapeutic potential
Gram-negative bacteria	*Salmonella* *Typhimurium*	Hijacks host SUMOylation to modify endocytic trafficking proteins	SUMO1 modification of Rab7 at K130 Blocks SCV maturation into lysosomes (75%reduction in LAMP1 + vesicles) Forms protective replicative niche	Rab7 SUMOylation inhibitors (e.g., TAK-981 derivatives)
	*Shigella flexneri*	Disrupts SUMOylation to suppress immune signaling	Secretes effector IpaH4.5 to degrade Ubc9 Disassembles PML nuclear bodies (80% size reduction in IFN-β)	IpaH4.5-Ubc9 interaction blockers
Gram-positive bacteria	*Staphylococcus aureus*	Globally reduces host SUMOylation	Secreted phosphatase PtpA dephosphorylates Ubc9 (ser68) Impairs SUMOylation of NF-κB/STAT1 Reduces pro-inflammatory cytokines (70% decrease in TNF-α/IL-6)	PtpA inhibitors Ubc9 phosphorylation activators
	*Listeria monocytogenes*	Induces degradation of SUMO machinery	LLO toxin actives HUWE1 E3 ligase Mediates K48-ubiquitination of Ubc9 (half-life 8 h → 1.5 h) Suppresses inflammasome activation (60% reduced caspas-1 activity)	LLO-HUWE1 interaction inhibitors
Obligate intracellular bacteria	*Ehrlichia chaffeensis*	Utilizes host SUMOylation to modify bacterial effectors	Ank200 SUMO1-modified at K152/K215 Bind AT-rich chromatin regions Upregulates nucleotide synthesis genes (3.5-fold DHFR increase) Suppresses immune genes (5-fold IFN-γ reduction)	Ank200 nuclear localization signal blockers

Gram-negative bacteria: modulating vesicle trafficking and immune signaling Gram-negative bacteria, such as *Salmonella* and *Shigella*, often target host SUMOylation to manipulate vesicle trafficking and immune signaling pathways. For example, *Salmonella* *Typhimurium* exploits the host SUMOylation system to modify RAB7, a small GTPase involved in endocytic trafficking. SUMOylation of RAB7 alters its function, preventing the maturation of *Salmonella*-containing vacuoles (SCVs) into degradative compartments and instead stabilizing them as protective niches for bacterial replication (Chandrasekhar et al., 2023). Similarly, *Shigella* has been shown to interfere with host SUMOylation to modulate immune signaling, thereby evading detection and promoting its survival (Lapaquette et al., 2017). These examples highlight how Gram-negative bacteria co-opt host SUMOylation to disrupt cellular processes and create a favorable environment for infection. In contrast, Gram-positive bacteria, such as *S. aureus* and *L. monocytogenes*, often employ strategies that directly reduce host SUMOylation levels to impair immune responses. *L. monocytogenes*, for instance, secretes the virulence factor *Listeriolysin* O (LLO), which induces the degradation of Ubc9, the sole E2 conjugating enzyme required for SUMOylation (Zhuang et al., 2024). This global reduction in host SUMOylation dampens immune signaling pathways, such as the activation of pro-inflammatory cytokines, and disrupts cellular stress responses, creating a more permissive environment for bacterial proliferation. Similarly, *S. aureus* has been shown to interfere with host SUMOylation to evade immune detection (Youssouf et al., 2021), further underscoring the importance of this strategy for Gram-positive pathogens.

Obligate intracellular bacteria, such as *E. chaffeensis*, utilize SUMOylation to modulate host gene expression and promote their replication within specific cellular niches. For example, *E. chaffeensis* relies on the SUMOylation of its Ank200 protein, which interacts with host chromatin to alter gene expression patterns (Zhu et al., 2009). This interaction suppresses host immune responses while upregulating genes that support bacterial replication, such as those involved in nutrient acquisition and energy production. By hijacking host SUMOylation pathways, obligate intracellular bacteria create a tailored environment that supports their survival and proliferation.

The distinct strategies employed by Gram-negative, Gram-positive, and obligate intracellular bacteria to exploit host SUMOylation highlight the versatility of bacterial pathogens in manipulating host cellular processes. These differences also present opportunities for developing targeted therapies. For instance, inhibiting the SUMOylation of specific host proteins, such as RAB7 in *Salmonella* infections or Ubc9 in *Listeria* infections, could disrupt bacterial replication and enhance host immune responses. Similarly, targeting bacterial effectors that interact with host SUMOylation machinery, such as Ank200 in *E. chaffeensis*, could provide a novel approach to combat infections caused by obligate intracellular pathogens. Understanding these pathogen-specific strategies is crucial for designing effective therapeutic interventions.

## 4 SUMOylation and bacterial pathogenesis mechanisms

Post-translational modifications (PTMs) play a pivotal role in regulating protein function, localization, and stability, thereby influencing cellular processes and disease mechanisms (Geiss-Friedlander and Melchior, 2007). Among these modifications, SUMOylation—the covalent attachment of small ubiquitin-like modifier (SUMO) proteins to target substrates—has emerged as a critical regulatory mechanism in both eukaryotic and prokaryotic systems. In the context of bacterial pathogenesis, SUMOylation has been shown to significantly influence bacterial pathogenicity by modulating the expression of virulence factors and altering host-pathogen interactions (Ribet and Cossart, 2010a,b). This chapter explores the intricate role of SUMOylation in bacterial pathogenesis, highlighting its impact on bacterial survival, immune evasion, and the manipulation of host cellular machinery.

### 4.1 Influence of host SUMOylation on bacterial virulence factor expression

The dynamic interplay between host cellular processes and bacterial pathogens is a cornerstone of infectious disease biology. Among the myriad of host post-translational modifications, SUMOylation has emerged as a critical regulatory mechanism that can influence bacterial virulence. Host SUMOylation, a process involving the covalent attachment of SUMO (small ubiquitin-like modifier) proteins to target substrates, plays a pivotal role in modulating cellular signaling pathways, gene expression, and protein stability (Hay, 2005; Geiss-Friedlander and Melchior, 2007). Intriguingly, recent studies have revealed that host SUMOylation can also regulate the expression of bacterial virulence genes (Ribet and Cossart, 2010a,b). Changes in the host’s SUMOylation status may serve as a signal for bacteria to upregulate or downregulate specific virulence factors, thereby adapting to the host environment and enhancing their pathogenicity (Praefcke et al., 2012).

A striking example of this phenomenon is observed in *Shigella*, a Gram-negative bacterium responsible for bacillary dysentery. *Shigella* has evolved sophisticated mechanisms to manipulate host cellular processes, including calcium signaling and calpain activity, to inhibit host SUMOylation (Ashida et al., 2015). This inhibition of SUMOylation may indirectly affect bacterial gene expression, enabling *Shigella* to fine-tune its virulence strategies in response to the host’s cellular state (Ribet and Cossart, 2015). By disrupting host SUMOylation, *Shigella* not only evades immune detection but also creates a favorable intracellular niche for its survival and replication (Hickey et al., 2012). This example underscores the broader implications of host SUMOylation in bacterial pathogenesis, highlighting its role as a regulatory node that bacteria can exploit to enhance their infectivity.

### 4.2 Bacterial modulation of SUMOylation to enhance pathogenicity

The ability of bacterial pathogens to manipulate host cellular processes is a hallmark of their evolutionary adaptation and a key factor in their success as infectious agents. Among the host post-translational modifications targeted by bacteria, SUMOylation has emerged as a critical regulatory mechanism that pathogens exploit to enhance their virulence. By altering host SUMOylation, bacteria can disrupt immune signaling, evade host defenses, and create a favorable environment for their survival and replication (Hay, 2005; Geiss-Friedlander and Melchior, 2007). This section explores how specific bacterial pathogens, such as *Brucella melitensis* and *K. pneumoniae*, manipulate host SUMOylation to enhance their pathogenicity.

*Brucella melitensis*, the causative agent of brucellosis, provides a compelling example of bacterial interference with host SUMOylation. This pathogen interacts directly with host SUMO1 and Ubc9, the E2 conjugating enzyme essential for SUMOylation (Ribet and Cossart, 2010a,b). By modulating the SUMOylation of proteins involved in immune responses, *B. melitensis* effectively evades host defenses and establishes a persistent infection (de Jong and Tsolis, 2012; Yi et al., 2019; Ma et al., 2023). This interaction highlights the strategic exploitation of host SUMOylation pathways by bacterial pathogens to subvert immune surveillance and promote their survival.

Similarly, *K. pneumoniae*, a leading cause of hospital-acquired infections, has been shown to reduce host SUMOylation as a means of limiting defense responses (Paczosa and Mecsas, 2016; Sá-Pessoa et al., 2020). By decreasing the SUMOylation of proteins involved in immune signaling, *K. pneumoniae* suppresses cytokine production and other defense mechanisms, thereby enhancing its virulence (Hickey et al., 2012). This reduction in host SUMOylation not only dampens the immune response but also creates an environment conducive to bacterial proliferation and dissemination.

These examples underscore the diverse strategies employed by bacterial pathogens to manipulate host SUMOylation. By targeting this critical post-translational modification, bacteria can disrupt host cellular processes, evade immune detection, and enhance their pathogenicity.

### 4.3 SUMOylation of host proteins by bacterial effectors

In the intricate battle between bacterial pathogens and their hosts, the manipulation of host cellular processes is a key strategy for bacterial survival and proliferation. Among the various mechanisms employed, some bacterial pathogens have evolved the ability to directly induce the SUMOylation of host proteins through the action of specialized effector molecules (Hay, 2005; Geiss-Friedlander and Melchior, 2007). These effectors hijack the host SUMOylation machinery to modify specific host proteins, altering their function in ways that benefit the pathogen. This section explores how bacterial effectors exploit the host’s SUMOylation pathways, using *E. chaffeensis* as a prime example, and discusses the broader implications of this strategy for bacterial pathogenesis.

*Ehrlichia chaffeensis*, the causative agent of human monocytic ehrlichiosis, provides a striking example of how bacterial effectors can manipulate host SUMOylation. This pathogen secretes effector proteins that actively exploit host SUMOylation pathways to mediate interactions between bacterial and host proteins (Dunphy et al., 2014; Pittner et al., 2023). By inducing the SUMOylation of specific host proteins, *E. chaffeensis* disrupts normal cellular processes, such as immune signaling and apoptosis, creating an environment that facilitates its survival and replication (Ribet and Cossart, 2010a,b). This manipulation of host SUMOylation not only aids in immune evasion but also promotes the establishment of a persistent infection.

The SUMOylation of host proteins by bacterial effectors can have profound effects on cellular function. For example, SUMOylation can alter the stability, localization, or activity of host proteins involved in critical pathways such as inflammation, cell cycle regulation, and stress responses (Hickey et al., 2012; Praefcke et al., 2012). By targeting these pathways, bacterial pathogens can suppress host defenses, promote intracellular survival, and enhance their virulence. This strategy highlights the versatility of SUMOylation as a tool for bacterial manipulation of host cells.

The ability of bacterial effectors to directly induce host SUMOylation underscores the complexity of host-pathogen interactions and the sophistication of bacterial virulence mechanisms. Understanding these processes not only sheds light on the molecular basis of bacterial infections but also identifies potential therapeutic targets for disrupting pathogen-induced SUMOylation and restoring host cellular function.

### 4.4 Role of SUMOylation in critical steps of pathogenesis

SUMOylation plays a critical role in key pathogenic steps, including biofilm formation, invasion, and immune evasion. In biofilm formation, SUMOylation influences the expression of genes involved in this process, affecting bacterial adherence and resistance to antimicrobials (Hendriks and Vertegaal, 2016; López-Jiménez et al., 2024). During invasion, modulation of SUMOylation can alter cytoskeletal dynamics, enhancing bacterial entry into host cells, as seen in *S. flexneri* infection, where changes in SUMOylation lead to altered actin polymerization and membrane ruffling (Lapaquette et al., 2017; Pittner et al., 2023; Zhou et al., 2025). Additionally, by altering the SUMOylation of immune signaling proteins, bacteria can evade detection and destruction by the host immune system, further facilitating their survival and pathogenicity (Srikanth and Verma, 2017).

## 5 SUMOylation and host antibacterial defense

While bacterial pathogens exploit SUMOylation to enhance their virulence, the host also employs SUMOylation as a critical defense mechanism to counteract infections. This dual role of SUMOylation underscores its importance in the intricate interplay between pathogens and the host immune system (Zhou et al., 2025). In this section, we explore how the host utilizes SUMOylation to resist bacterial infections, enhance immune responses, and limit bacterial proliferation and spread.

### 5.1 Host utilization of SUMOylation to enhance immune responses and limit infections

The host leverages SUMOylation to strengthen its immune defenses by modifying key proteins involved in immune signaling and response (Wilson, 2012). For instance, SUMOylation stabilizes transcription factors such as NF-κB and STATs, which are central to the regulation of immune responses. By promoting the expression of antimicrobial peptides and pro-inflammatory cytokines, SUMOylation enhances the host’s ability to combat bacterial infections (Hendriks and Vertegaal, 2016). This stabilization ensures a robust and sustained immune response, which is critical for controlling pathogen proliferation.

In addition to stabilizing transcription factors, SUMOylation also enhances the activity of pattern recognition receptors (PRRs) and signaling molecules, which are essential for detecting and responding to bacterial infections. A notable example is the SUMOylation of RIG-I, a cytosolic receptor primarily known for its role in viral RNA recognition. SUMOylation of RIG-I increases its ability to induce interferon production, a response that not only targets viruses but also exerts antibacterial effects (Zhou et al., 2017). This cross-protective mechanism highlights the versatility of SUMOylation in bolstering the host’s immune defenses against diverse pathogens.

### 5.2 Limitation of bacterial proliferation and activation of host defense mechanisms

SUMOylation plays a pivotal role in restricting bacterial proliferation by promoting cellular processes such as autophagy and apoptosis. For example, SUMOylation of autophagy-related proteins enhances the formation of autophagosomes, which sequester and degrade intracellular bacteria, thereby limiting their survival (Dupont et al., 2010). Additionally, SUMOylation of the tumor suppressor protein p53 can induce apoptosis in infected cells, preventing the spread of bacteria to neighboring tissues and containing the infection (Dupont et al., 2010). These mechanisms demonstrate how SUMOylation contributes to the host’s ability to control and eliminate bacterial pathogens.

Furthermore, SUMOylation is integral to the activation of several host defense mechanisms, including autophagy and inflammatory signaling (K et al., 2021). For instance, SUMOylation of autophagy proteins like LC3 facilitates the clearance of intracellular pathogens, enhancing the host’s ability to eliminate infections (Dupont et al., 2010). Moreover, SUMOylation modulates the activity of inflammasomes and other components of the inflammatory response, ensuring a robust and coordinated immune reaction to bacterial infections (Hendriks and Vertegaal, 2016). These processes collectively enhance the host’s capacity to detect, respond to, and eliminate invading pathogens.

The host’s utilization of SUMOylation as a defense mechanism highlights its dual role in the host-pathogen interaction. While bacteria manipulate SUMOylation to enhance their virulence, the host counters by leveraging SUMOylation to strengthen immune responses, limit bacterial proliferation, and activate critical defense pathways. Understanding these mechanisms provides valuable insights into the molecular basis of host defense and opens new avenues for therapeutic strategies aimed at enhancing SUMOylation-mediated immune responses.

## 6 Future research directions in SUMOylation and bacterial infection

### 6.1 Elucidating pathogen-specific SUMOylation networks through multi-omics integration

While the role of SUMOylation in bacterial infections is increasingly recognized, most studies have focused on model pathogens (e.g., *Salmonella*, *Listeria*), leaving significant gaps in understanding how emerging and drug-resistant pathogens like *A. baumannii* or *M. abscessus* manipulate host SUMOylation. To address these limitations, we propose an integrated research strategy combining cutting-edge technologies and translational applications.

First, spatiotemporal sumoylome profiling, leveraging single-cell SUMO-proteomics (e.g., SUMO-APEX2 labeling) and spatial transcriptomics, can map SUMOylation dynamics in *A. baumannii* -infected lung tissues at high resolution. A study in 2024 Nature Methods demonstrated this approach’s capability to resolve host-pathogen SUMOylation crosstalk at 5-μm resolution, offering unprecedented insights into infection-specific SUMOylation networks (Sahin et al., 2022). Second, CRISPR-SUMO screens using dCas9-SUMO2 fusions can systematically perturb SUMOylation sites genome-wide in infected macrophages, identifying novel regulatory checkpoints. For instance, Kim et al. (2021) suggested that sumoylated mitochondrial proteins may restrict *M. tuberculosis* growth, highlighting potential targets for host-directed therapies (Huang et al., 2024).

These findings could enable pathogen-specific SUMO-targeted therapies. For example, since *A. baumannii* biofilm formation relies on host SUMO3 (but not SUMO1), isoform-selective inhibitors could minimize off-target effects while maintaining therapeutic efficacy (Huang et al., 2024). Such precision approaches may revolutionize treatment strategies against recalcitrant infections.

### 6.2 AI-driven design of SUMO-targeted antimicrobials

The development of SUMOylation-targeted antimicrobials faces significant challenges, particularly regarding specificity and safety. Current broad-spectrum SUMO inhibitors like TAK-981, which target the SUMO-E1 enzyme, often lead to undesirable toxicity due to their indiscriminate action on host cellular processes (Huang et al., 2024). Moreover, the field lacks pathogen-specific inhibitors that can precisely disrupt bacterial effector proteins’ manipulation of host SUMOylation systems without affecting normal cellular functions (Huang et al., 2024).

To address these challenges, AI-driven approaches are being increasingly utilized to identify and develop pathogen-specific SUMO inhibitors. These methods leverage the power of computational tools and machine learning algorithms to predict and optimize interactions between bacterial virulence factors and host SUMO proteins. For instance, AlphaFold-Multimer, a computational tool primarily used for structure prediction, has been instrumental in mapping high-affinity binding interfaces between bacterial effectors and host SUMO proteins. It is important to note that while AlphaFold-Multimer provides valuable structural predictions, experimental validation remains essential to confirm the structural accuracy and functional relevance of these prediction (Strom and Luck, 2025).

In addition to structure prediction, AI models such as DiffDock are being employed to simulate and optimize small molecule interactions with SUMO-effector complexes. A landmark study published in Science in 2024 reported that these AI-driven methods achieved a remarkable 50% hit rate in identifying compounds that specifically target the *Salmonella* SifA-Ubc9 interaction interface (Mirabello et al., 2024). This success highlights the potential of AI in accelerating the discovery of novel antimicrobial agents.

The translation of these computational breakthroughs into clinical applications is already underway. Building on the framework of the NCT05532826 trial, next-generation Phase I/II clinical studies are evaluating AI-designed SUMO inhibitors (e.g., AUR-SUMO1i) for treating carbapenem-resistant infections (Huang et al., 2024). These trials incorporate innovative monitoring strategies, such as tracking serum levels of SUMOylated HMGB1, to assess therapeutic efficacy and potential off-target effects in real-time. This biomarker approach allows for dynamic treatment adjustments, potentially overcoming the toxicity limitations of earlier generation SUMO inhibitors.

**Figure 3:**
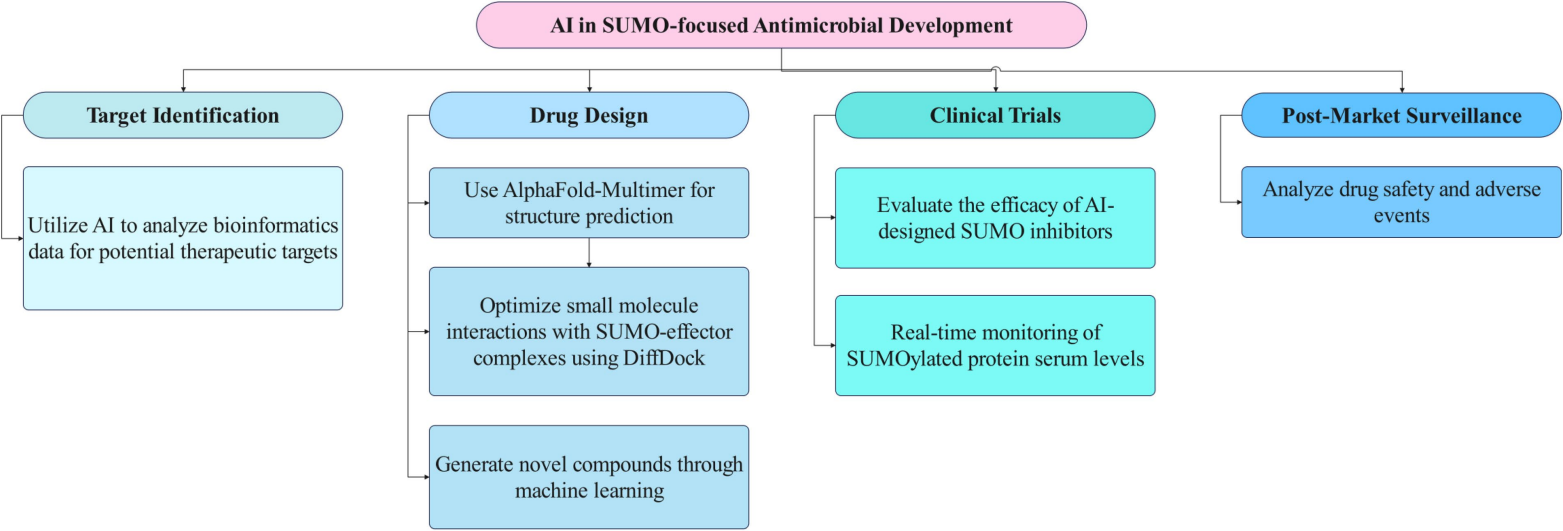
The flowchart illustrates the interface between AI technologies and SUMO-focused antimicrobial strategies. This diagram outlines the stages of AI- and SUMO-focused antimicrobial development. It starts with identifying targets using AI to analyze genomic data. Next, drug design involves structure prediction and optimizing compounds with AI. Clinical trials assess the efficacy of these inhibitors, followed by post-market surveillance to monitor drug safety and effectiveness.

Furthermore, AI-driven strategies are being expanded to target other critical pathogen-SUMO interactions, including those employed by ESKAPE pathogens and emerging antimicrobial-resistant strains (Huang et al., 2024). By combining high-resolution structural prediction with machine learning-based compound generation, researchers are developing a new class of precision antimicrobials that specifically disrupt pathogenic manipulation of host SUMOylation while preserving essential host functions. This shift from broad inhibition to targeted interference represents a promising avenue for overcoming current limitations in antimicrobial development (Figure 3).

In summary, AI technologies are playing a crucial role in advancing the development of SUMO-targeted antimicrobials. By focusing on identifying host-pathogen SUMO interfaces and developing pathogen-specific inhibitors, these approaches hold the potential to revolutionize the treatment of bacterial infections and combat antimicrobial resistance.

### 6.3 Global SUMOylation surveillance for antimicrobial resistance (AMR)

In the context of the growing threat of antimicrobial resistance (AMR), the need for global surveillance and intervention of drug resistance has become increasingly urgent. To address this challenge, a vision called the “SUMO-AMR Atlas” has been proposed, aiming to integrate clinical sumoylomes (Sumoylated proteomes) with AI-based predictive models (Bilal et al., 2025). Specifically, the clinical sumoylome data will be sourced from the WHO’s Global Antimicrobial Resistance and Use Surveillance System (GLASS) network (Bilal et al., 2025). By analyzing the correlation between SUMOylation patterns (e.g., hyper-SUMOylated STAT1) and resistance phenotypes, this initiative offers a new perspective for AMR research. Additionally, AI predictive models will be employed to forecast the evolution of resistance based on mutations in bacterial SUMO effectors (e.g., *E. coli* IpaH9.8-K215R) (Bilal et al., 2025).

To realize this vision, the National Institutes of Health (NIH) has launched the “SUMOnet” pilot project (2025–2030), which plans to profile 10,000 bacterial isolates using SUMO-targeted nanopore sequencing (Cesaro et al., 2025). This project will leverage advanced nanopore sequencing technology to conduct high-throughput analysis of bacterial SUMOylation states, thereby providing a wealth of data to support the construction of the SUMO-AMR Atlas. By integrating the clinical data from the GLASS network with the experimental data from the SUMOnet project, the SUMO-AMR Atlas is expected to offer powerful tools and strategies for global AMR surveillance and intervention (Cesaro et al., 2025).

## 7 Conclusion

**Figure 4:**
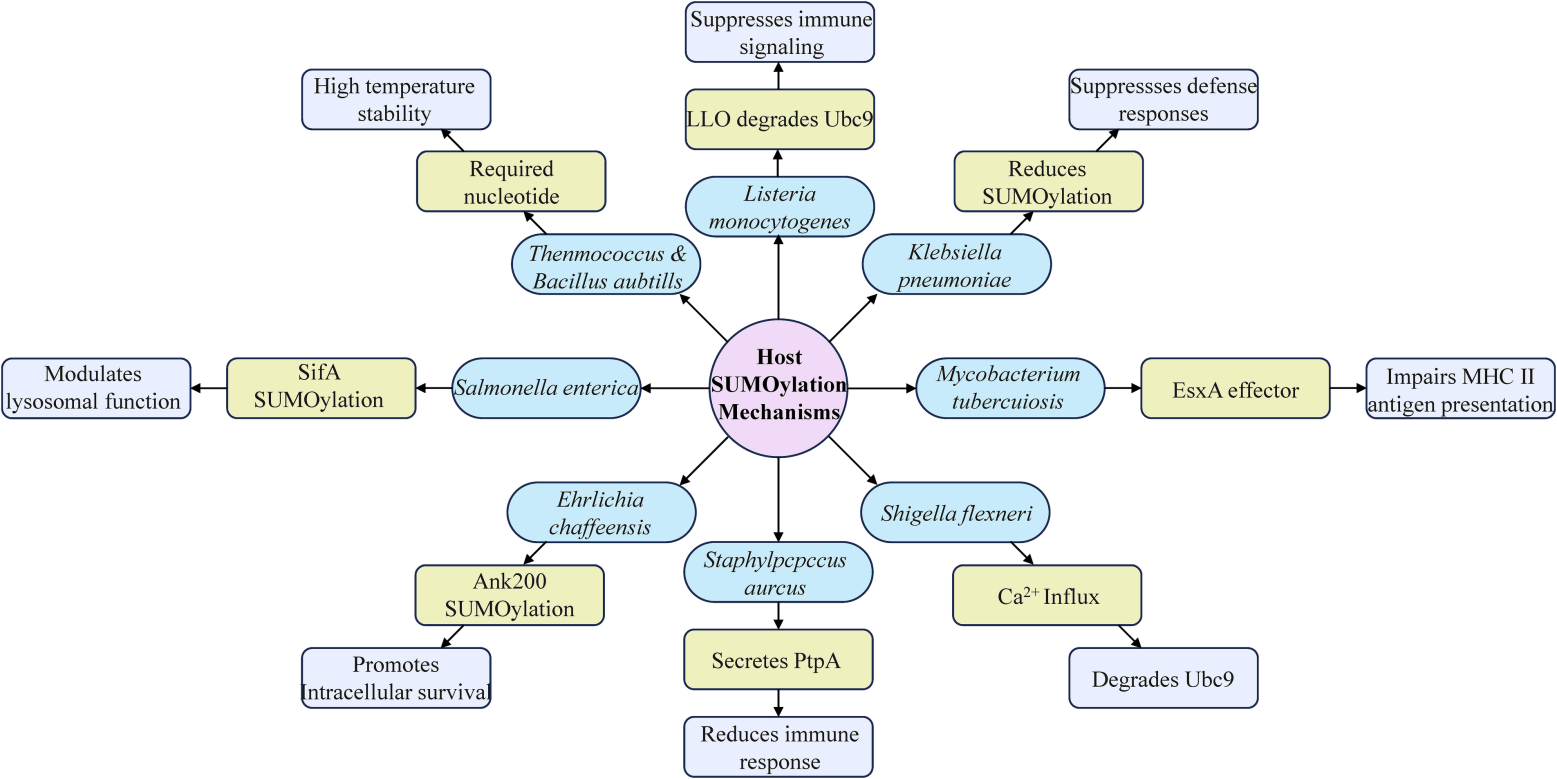
Overview of host SUMOylation in bacterial infection and its immunomodulatory roles. This diagram illustrates how various bacteria utilize SUMOylation to modulate host immune responses. It highlights key mechanisms by which bacteria such as *Salmonella*, *Listeria monocytogenes, Klebsiella pneumoniae*, *Mycobacterium tuberculosis*, *Ehrlichia chaffeensis*, *Staphylococcus aureus*, and *Shigella flexneri* manipulate host cellular processes to evade or suppress immune defenses.

The intricate interplay between SUMOylation and bacterial infections highlights the multifaceted roles that this post-translational modification plays in both host defense and bacterial pathogenicity. As a critical regulatory mechanism, SUMOylation modulates a wide range of cellular processes that are essential for maintaining homeostasis and mounting effective immune responses against invading pathogens. However, bacterial pathogens have evolved sophisticated strategies to hijack and manipulate host SUMOylation machinery for their own benefit, thereby enhancing their virulence and evading host defenses (Figure 4).

This review has provided a comprehensive overview of the current understanding of SUMOylation in host-pathogen interactions, emphasizing the dual roles of SUMOylation in bacterial infections. We have discussed how SUMOylation regulates host immune responses, from enhancing the stability and activity of key immune signaling molecules to promoting antimicrobial mechanisms such as autophagy and apoptosis. Conversely, we have also explored the various ways in which bacterial pathogens exploit SUMOylation to overcome host immunity, manipulate cellular processes, and create a favorable environment for their survival and replication.

Despite significant progress in this field, several key challenges remain unresolved. One of the most critical issues is the difficulty in selectively targeting pathogen-specific SUMO manipulations without perturbing host homeostasis. Current broad-spectrum SUMO inhibitors, such as TAK-981, often lead to undesirable toxicity due to their indiscriminate action on host cellular processes. Developing pathogen-specific inhibitors that can precisely disrupt bacterial effector proteins’ manipulation of host SUMOylation systems without affecting normal cellular functions is a significant challenge that requires further research.

Another major gap is the limited availability of global SUMOylome datasets for certain pathogens. For instance, comprehensive SUMOylation profiles for emerging and drug-resistant pathogens like *A. baumannii* or *M. abscessus* are still lacking. These datasets are crucial for understanding the full extent of SUMOylation changes during infection and identifying potential therapeutic targets.

Future research in this field should focus on addressing these challenges. Concrete future directions include developing high-resolution SUMOylation interaction maps by leveraging advanced proteomic and genomic techniques to generate detailed maps of SUMOylation interactions in both host and pathogen proteins. This will provide a comprehensive understanding of the dynamic changes in SUMOylation during infection and identify key nodes for therapeutic intervention. Additionally, developing pathogen-specific SUMO inhibitors is a critical next step. This will require a combination of structural biology, computational modeling, and high-throughput screening to identify and optimize inhibitors that selectively target pathogen-specific SUMOylation pathways without disrupting host homeostasis. Finally, conducting host-directed therapy trials is essential. These trials should evaluate the efficacy of SUMO-targeted therapies in treating bacterial infections and incorporate innovative monitoring strategies, such as tracking serum levels of SUMOylated proteins, to assess therapeutic efficacy and potential off-target effects.

In conclusion, while significant progress has been made in understanding the role of SUMOylation in bacterial infections, substantial gaps in our knowledge remain. Addressing these gaps through innovative research approaches and interdisciplinary collaborations will be essential for developing effective strategies to combat bacterial infections and antimicrobial resistance. The insights gained from these efforts will not only enhance our understanding of host-pathogen interactions but also pave the way for the development of novel anti-infective therapies that target the intricate interplay between SUMOylation and bacterial pathogenesis.

## References

[ref1] Anang V. Singh A. Kumar Rana A. Saraswati S. S. K. Bandyopadhyay U. Verma C.. (2023). Mycobacteria modulate SUMOylation to suppresses protective responses in dendritic cells. PLoS One 18:e0283448. doi: 10.1371/journal.pone.0283448, PMID: 37773921 PMC10540951

[ref2] Ashida H. Mimuro H. Sasakawa C. (2015). *Shigella* manipulates host immune responses by delivering effector proteins with specific roles. Front. Immunol. 6:219. doi: 10.3389/fimmu.2015.00219, PMID: 25999954 PMC4423471

[ref3] Beyer A. R. Truchan H. K. May L. J. Walker N. J. Borjesson D. L. Carlyon J. A. (2015). The *Anaplasma phagocytophilum* effector AmpA hijacks host cell SUMOylation. Cell. Microbiol. 17, 504–519. doi: 10.1111/cmi.12380, PMID: 25308709 PMC4664186

[ref4] Bhachoo J. S. Garvin A. J. (2024). SUMO and the DNA damage response. Biochem. Soc. Trans. 52, 773–792. doi: 10.1042/BST20230862, PMID: 38629643 PMC11088926

[ref5] Bilal H. Khan M. N. Khan S. Shafiq M. Fang W. Khan R. U.. (2025). The role of artificial intelligence and machine learning in predicting and combating antimicrobial resistance. Comput. Struct. Biotechnol. J. 27, 423–439. doi: 10.1016/j.csbj.2025.01.006, PMID: 39906157 PMC11791014

[ref6] Cesaro A. Hoffman S. C. das P. de la Fuente-Nunez C. (2025). Challenges and applications of artificial intelligence in infectious diseases and antimicrobial resistance. NPJ Antimicrob Resist 3:2. doi: 10.1038/s44259-024-00068-x, PMID: 39843587 PMC11721440

[ref7] Chandrasekhar H. Mohapatra G. Kajal K. Singh M. Walia K. Rana S.. (2023). SifA SUMOylation governs *Salmonella Typhimurium* intracellular survival via modulation of lysosomal function. PLoS Pathog. 19:e1011686. doi: 10.1371/journal.ppat.1011686, PMID: 37773952 PMC10566704

[ref8] de Jong M. F. Tsolis R. M. (2012). Brucellosis and type IV secretion. Future Microbiol. 7, 47–58. doi: 10.2217/fmb.11.136, PMID: 22191446

[ref9] Dunphy P. S. Luo T. McBride J. W. (2014). *Ehrlichia chaffeensis* exploits host SUMOylation pathways to mediate effector-host interactions and promote intracellular survival. Infect. Immun. 82, 4154–4168. doi: 10.1128/IAI.01984-14, PMID: 25047847 PMC4187855

[ref10] Dupont N. Temime-Smaali N. Lafont F. (2010). How ubiquitination and autophagy participate in the regulation of the cell response to bacterial infection. Biol. Cell. 102, 621–634. doi: 10.1042/BC20100101, PMID: 21077843 PMC2975374

[ref11] Ekstedt R. D. (1974). Immune response to surface antigens of *Staphylococcus aureus* and their role in resistance to staphylococcal disease. Ann. N. Y. Acad. Sci. 236, 203–220. 4214129 10.1111/j.1749-6632.1974.tb41492.x

[ref12] Flotho A. Melchior F. (2013). Sumoylation: a regulatory protein modification in health and disease. Annu. Rev. Biochem. 82, 357–385. doi: 10.1146/annurev-biochem-061909-093311, PMID: 23746258

[ref13] Fritah S. Lhocine N. Golebiowski F. Mounier J. Andrieux A. Jouvion G.. (2014). Sumoylation controls host anti-bacterial response to the gut invasive pathogen *Shigella flexneri*. EMBO Rep. 15, 965–972. doi: 10.15252/embr.201338386, PMID: 25097252 PMC4198040

[ref14] Gareau J. R. Lima C. D. (2010). The SUMO pathway: emerging mechanisms that shape specificity, conjugation and recognition. Nat. Rev. Mol. Cell Biol. 11, 861–871. doi: 10.1038/nrm3011, PMID: 21102611 PMC3079294

[ref15] Geiss-Friedlander R. Melchior F. (2007). Concepts in sumoylation: a decade on. Nat. Rev. Mol. Cell Biol. 8, 947–956. doi: 10.1038/nrm2293, PMID: 18000527

[ref16] Hay R. T. (2005). SUMO: a history of modification. Mol. Cell 18, 1–12. doi: 10.1016/j.molcel.2005.03.012, PMID: 15808504

[ref17] Hendriks I. A. Vertegaal A. C. O. (2016). A comprehensive compilation of SUMO proteomics. Nat. Rev. Mol. Cell Biol. 17, 581–595. doi: 10.1038/nrm.2016.81, PMID: 27435506

[ref18] Hickey C. M. Wilson N. R. Hochstrasser M. (2012). Function and regulation of SUMO proteases. Nat. Rev. Mol. Cell Biol. 13, 755–766. doi: 10.1038/nrm3478, PMID: 23175280 PMC3668692

[ref19] Hu M. M. Shu H. B. (2020). Innate immune response to cytoplasmic DNA: mechanisms and diseases. Annu. Rev. Immunol. 38, 79–98. doi: 10.1146/annurev-immunol-070119-115052, PMID: 31800327

[ref20] Huang C. H. Yang T. T. Lin K. I. (2024). Mechanisms and functions of SUMOylation in health and disease: a review focusing on immune cells. J. Biomed. Sci. 31:16. doi: 10.1186/s12929-024-01003-y, PMID: 38280996 PMC10821541

[ref21] K S. T. Joshi G. Arya P. Mahajan V. Chaturvedi A. Mishra R. K. (2021). SUMO and SUMOylation pathway at the forefront of host immune response. Front. Cell Dev. Biol. 9:681057. doi: 10.3389/fcell.2021.681057, PMID: 34336833 PMC8316833

[ref22] Kaufmann S. H. E. Dorhoi A. Hotchkiss R. S. Bartenschlager R. (2018). Host-directed therapies for bacterial and viral infections. Nat. Rev. Drug Discov. 17, 35–56. doi: 10.1038/nrd.2017.162, PMID: 28935918 PMC7097079

[ref23] Kim C. Juncker M. Reed R. Haas A. Guidry J. Matunis M.. (2021). SUMOylation of mitofusins: a potential mechanism for perinuclear mitochondrial congression in cells treated with mitochondrial stressors. Biochim. Biophys. Acta Mol. basis Dis. 1867:166104. doi: 10.1016/j.bbadis.2021.166104, PMID: 33617988 PMC8261867

[ref24] Lapaquette P. Fritah S. Lhocine N. Andrieux A. Nigro G. Mounier J.. (2017). Shigella entry unveils a calcium/calpain-dependent mechanism for inhibiting sumoylation. eLife 6:7444. doi: 10.7554/eLife.27444, PMID: 29231810 PMC5745084

[ref25] Li J. Lam W. W. L. Lai T. W. Au S. W. N. (2017). Degradation of nuclear Ubc 9 induced by listeriolysin O is dependent on K(+) efflux. Biochem. Biophys. Res. Commun. 493, 1115–1121. doi: 10.1016/j.bbrc.2017.09.051, PMID: 28911869

[ref26] Liu Y. Zheng Z. Shu B. Meng J. Zhang Y. Zheng C.. (2016). SUMO modification stabilizes enterovirus 71 polymerase 3D to facilitate viral replication. J. Virol. 90, 10472–10485. doi: 10.1128/JVI.01756-16, PMID: 27630238 PMC5110190

[ref27] López-Jiménez A. T. Özbaykal Güler G. Mostowy S. (2024). The great escape: a Shigella effector unlocks the septin cage. Nat. Commun. 15:4104. doi: 10.1038/s41467-024-48208-1, PMID: 38750009 PMC11096336

[ref28] Ma X. Zhao C. Xu Y. Zhang H. (2023). Roles of host SUMOylation in bacterial pathogenesis. Infect. Immun. 91:e0028323. doi: 10.1128/iai.00283-23, PMID: 37725062 PMC10580907

[ref29] Mirabello C. Wallner B. Nystedt B. Azinas S. Carroni M. (2024). Unmasking AlphaFold to integrate experiments and predictions in multimeric complexes. Nat. Commun. 15:8724. doi: 10.1038/s41467-024-52951-w, PMID: 39379372 PMC11461844

[ref30] Mohapatra G. Gaur P. Mujagond P. Singh M. Rana S. Pratap S.. (2019). A SUMOylation-dependent switch of RAB7 governs intracellular life and pathogenesis of *Salmonella Typhimurium*. J. Cell Sci. 132:2612. doi: 10.1242/jcs.222612, PMID: 30510112

[ref31] Paczosa M. K. Mecsas J. (2016). *Klebsiella pneumoniae*: going on the offense with a strong defense. Microbiol. Mol. Biol. Rev. 80, 629–661. doi: 10.1128/MMBR.00078-15, PMID: 27307579 PMC4981674

[ref32] Pittner N. A. Solomon R. N. Bui D. C. McBride J. W. (2023). Ehrlichia effector SLiM-icry: artifice of cellular subversion. Front. Cell. Infect. Microbiol. 13:1150758. doi: 10.3389/fcimb.2023.1150758, PMID: 36960039 PMC10028187

[ref33] Praefcke G. J. Praefcke G. J. K. Hofmann K. Dohmen R. J. (2012). SUMO playing tag with ubiquitin. Trends Biochem. Sci. 37, 23–31. doi: 10.1016/j.tibs.2011.09.002, PMID: 22018829

[ref34] Ribet D. Cossart P. (2010a). Pathogen-mediated posttranslational modifications: a re-emerging field. Cell 143, 694–702. doi: 10.1016/j.cell.2010.11.019, PMID: 21111231 PMC7112265

[ref35] Ribet D. Cossart P. (2010b). Post-translational modifications in host cells during bacterial infection. FEBS Lett. 584, 2748–2758. doi: 10.1016/j.febslet.2010.05.012, PMID: 20493189

[ref36] Ribet D. Cossart P. (2015). How bacterial pathogens colonize their hosts and invade deeper tissues. Microbes Infect. 17, 173–183. doi: 10.1016/j.micinf.2015.01.004, PMID: 25637951

[ref37] Ribet D. Hamon M. Gouin E. Nahori M. A. Impens F. Neyret-Kahn H.. (2010). *Listeria monocytogenes* impairs SUMOylation for efficient infection. Nature 464, 1192–1195. doi: 10.1038/nature08963, PMID: 20414307 PMC3627292

[ref38] Sahin U. de Thé H. Lallemand-Breitenbach V. (2022). Sumoylation in physiology, pathology and therapy. Cells 11:814. doi: 10.3390/cells11050814, PMID: 35269436 PMC8909597

[ref39] Sá-Pessoa J. Przybyszewska K. Vasconcelos F. N. Dumigan A. Frank C. G. Hobley L.. (2020). *Klebsiella pneumoniae* reduces SUMOylation to limit host defense responses. MBio 11:e01733–20. doi: 10.1128/mBio.01733-20, PMID: 32994335 PMC7527722

[ref40] Srikanth C. V. Verma S. (2017). Sumoylation as an integral mechanism in bacterial infection and disease progression. Adv. Exp. Med. Biol. 963, 389–408. doi: 10.1007/978-3-319-50044-7_22, PMID: 28197924

[ref41] Strom J. M. Luck K. (2025). Bias in, bias out – AlphaFold-Multimer and the structural complexity of protein interfaces. Curr. Opin. Struct. Biol. 91:103002. doi: 10.1016/j.sbi.2025.103002, PMID: 39938238

[ref42] Tang Z. Hecker C. M. Scheschonka A. Betz H. (2008). Protein interactions in the sumoylation cascade: lessons from X-ray structures. FEBS J. 275, 3003–3015. doi: 10.1111/j.1742-4658.2008.06459.x, PMID: 18492068

[ref43] Tian L. Zhou W. Wu X. Hu Z. Qiu L. Zhang H.. (2022). CTLs: killers of intracellular bacteria. Front. Cell. Infect. Microbiol. 12:967679. doi: 10.3389/fcimb.2022.967679, PMID: 36389159 PMC9645434

[ref44] Wilson V. G. (2012). Sumoylation at the host-pathogen interface. Biomol. Ther. 2, 203–227. doi: 10.3390/biom2020203, PMID: 23795346 PMC3685863

[ref45] Wimmer P. Schreiner S. Dobner T. (2012). Human pathogens and the host cell SUMOylation system. J. Virol. 86, 642–654. doi: 10.1128/JVI.06227-11, PMID: 22072786 PMC3255802

[ref46] Xu Y. Zhou P. Cheng S. Lu Q. Nowak K. Hopp A. K.. (2019). A bacterial effector reveals the V-ATPase-ATG16L1 Axis that initiates Xenophagy. Cell 178, 552–566.e20. doi: 10.1016/j.cell.2019.06.007, PMID: 31327526

[ref47] Yi J. Wang Y. Li Q. Zhang H. Shao Z. Deng X. Y.. (2019). Interaction between *Brucella melitensis* 16M and small ubiquitin-related modifier 1 and E2 conjugating enzyme 9 in mouse RAW264.7 macrophages. J. Vet. Sci. 20:e54. doi: 10.4142/jvs.2019.20.e54, PMID: 31565897 PMC6769333

[ref48] You J. Xu A. Wang Y. Tu G. Huang R. Wu S. (2025). The STING signaling pathways and bacterial infection. Apoptosis 30, 389–400. doi: 10.1007/s10495-024-02031-7, PMID: 39428409

[ref49] Youssouf N. Recasens-Zorzo C. Molle V. Bossis G. Soubeyran P. Gannoun-Zaki L. (2021). *Staphylococcus aureus* decreases SUMOylation host response to promote Intramacrophage survival. Int. J. Mol. Sci. 22:8108. doi: 10.3390/ijms22158108, PMID: 34360873 PMC8347835

[ref50] Zhao H. Zhang X. Zhang N. Zhu L. Lian H. (2025). The interplay between Salmonella and host: mechanisms and strategies for bacterial survival. Cell Insight 4:100237. doi: 10.1016/j.cellin.2025.100237, PMID: 40177681 PMC11964643

[ref51] Zhou Y. He C. Wang L. Ge B. (2017). Post-translational regulation of antiviral innate signaling. Eur. J. Immunol. 47, 1414–1426. doi: 10.1002/eji.201746959, PMID: 28744851 PMC7163624

[ref52] Zhou X. Zhang H. Wang Y. Wang D. Lin Z. Zhang Y.. (2025). *Shigella* effector IpaH1.4 subverts host E3 ligase RNF213 to evade antibacterial immunity. Nat. Commun. 16:3099. doi: 10.1038/s41467-025-58432-y, PMID: 40164614 PMC11958729

[ref53] Zhu B. Nethery K. A. Kuriakose J. A. Wakeel A. Zhang X. McBride J. W. (2009). Nuclear translocated *Ehrlichia chaffeensis* ankyrin protein interacts with a specific adenine-rich motif of host promoter and intronic Alu elements. Infect. Immun. 77, 4243–4255. doi: 10.1128/IAI.00376-09, PMID: 19651857 PMC2747939

[ref54] Zhu G. Tong N. Zhu Y. Wang L. Wang Q. (2025). The crosstalk between SUMOylation and immune system in host-pathogen interactions. Crit. Rev. Microbiol. 51, 164–186. doi: 10.1080/1040841X.2024.2339259, PMID: 38619159

[ref55] Zhuang Y. Fischer J. B. Nishanth G. Schlüter D. (2024). Cross-regulation of Listeria monocytogenes and the host ubiquitin system in listeriosis. Eur. J. Cell Biol. 103:151401. doi: 10.1016/j.ejcb.2024.151401, PMID: 38442571

